# Frequency-dependent climate sensitivity of sub-daily radial growth of tree stems at the dry Arctic treeline

**DOI:** 10.1093/treephys/tpag068

**Published:** 2026-05-19

**Authors:** Jan Tumajer, Håkan Grudd, Hana Kuželová, Jelena Lange, Václav Treml

**Affiliations:** Department of Physical Geography and Geoecology, Faculty of Science, Charles University, Albertov 6, Prague 12843, Czech Republic; Swedish Polar Research Secretariat, Abisko Scientific Research Station, Vetenskapens Väg 38, Abisko 981 07, Sweden; Department of Physical Geography and Geoecology, Faculty of Science, Charles University, Albertov 6, Prague 12843, Czech Republic; Department of Physical Geography and Geoecology, Faculty of Science, Charles University, Albertov 6, Prague 12843, Czech Republic; Institute of Botany and Landscape Ecology, Department of Biology, Faculty of Mathematics and Natural Sciences, University of Greifswald, Soldmannstraße 15, Greifswald 17487, Germany; Department of Physical Geography and Geoecology, Faculty of Science, Charles University, Albertov 6, Prague 12843, Czech Republic

**Keywords:** boreal forest, cambial activity, dendrometer, phenology, *Pinus sylvestris*, wood formation, xylogenesis

## Abstract

Radial growth of tree stems shows remarkable variability over the year but also within individual days. Understanding the frequency-dependent growth sensitivity, i.e., the shifting responses of wood formation to meteorological conditions between annual and daily temporal scales, is essential for predicting forest growth under future climates. However, this knowledge is limited for the cold Arctic margins of species distribution. To address this gap, we monitored intra-annual and sub-daily stem radius variation of *Pinus sylvestris* L. for 2 years using xylogenesis microsampling and dendrometers at the cold-dry treeline beyond the Arctic Circle near Abisko, Northern Sweden. Using linear statistics, cell growth modeling and wavelet transformation, we separated individual frequencies of stem oscillations, ranked their statistical importance and identified immediate and lagged meteorological drivers of stem radius increment. Radial growth of tree stems showed overlapping oscillations at annual and daily frequencies. On an annual scale, radial growth and cell production peaked during the warm summer months. However, within the summer, stem radius increment accelerated toward the cool, moist midnight hours and ceased during the day as the temperature and solar altitude increased. Accordingly, we identified two growth-optimal intervals with peak growth rates, jointly accounting for 68% of the total growth: (i) an air temperature of 8–16 °C with a vapor pressure deficit (VPD) of <0.2 kPa, and (ii) an air temperature of 4–16 °C and VPD = 0 kPa. While correlations between peak-summer growth rates and immediate temperature and VPD were negative, these correlations switched to positive when considering lagged meteorological variables preceding growth by up to 4 days. Our results suggest frequency-dependent shifts and lagged responses of radial growth to meteorological variables at the Arctic treeline, particularly for air temperature and humidity. We propose that the midday growth reduction during summer may help explain non-linear responses of northern boreal forests to recent climate warming.

## Introduction

Radial tree growth is crucial for carbon sequestration from the atmosphere into terrestrial ecosystems (Pan et al. 2011, Friend et al. 2019). In climatic zones with distinct seasons, the process of wood formation responsible for radial growth is periodically constrained by instantaneous meteorological conditions. For instance, wood formation is limited by low temperatures in cold environments such as high latitudes or elevations, resulting in high rates of radial stem growth during the short warm season (Rossi et al. 2006*b*, Dobbert et al. 2022, Lu et al. 2025) and in a tight coupling of latewood density series with instrumental records of summer temperatures (Grudd 2008, Björklund et al. 2023). In addition to the annual cycle, recent studies have shown systematic sub-daily variation of stem growth for various biomes including temperate (Zweifel et al. 2021), submontane (Ziaco and Biondi 2018), Mediterranean (Güney et al. 2020, Vieira et al. 2022) and tropical forests (Luse Belanganayi et al. 2024). In particular, radial growth tends to peak at night when air humidity is highest, resulting in a fragmentation of the growing season into discontinuous nocturnal episodes (Etzold et al. 2022) and leading to negative correlations of sub-daily stem growth rates with instant temperature (Tumajer et al. 2022, Plavcová et al. 2025). Consequently, radial growth is often more sensitive to variability of temperature and humidity during nighttime compared with daytime (Han et al. 2025). However, little is known about the diel cycle of stem growth at cold treeline sites where growth is primarily limited by low temperature (but see King et al. 2013, Flynn et al. 2025). Moreover, the summer diel amplitude of stem growth beyond the Arctic Circle and how the midnight sun affects climate-growth responses on a sub-daily scale have not yet been comprehensively elucidated.

The process of wood formation is coordinated by internal cues but also responds to specific meteorological thresholds that need to be surpassed (Buttò et al. 2025). As air temperature decreases, the mitotic cycle slows down and leads to a cessation of cambial activity below ~5 °C (Rossi et al. 2007, Treml et al. 2015). In addition to temperature, wood formation may be constrained by moisture availability: stem shrinkage has been reported during periods of low turgor pressure due to reduced cell division, lumen enlargement and increased tree water deficit (Cabon et al. 2020, Peters et al. 2021). Turgor pressure is determined by the water content inside the stem and reflects soil water potential, sapflow rate, stomatal conductance and solute concentrations driving osmotic flow (Pallardy 2008). Accordingly, water loss through transpiration during the day and refilling at night are plausible causes for the diel stem cycles observed in temperate climates with prevailingly nocturnal growth (Haasis 1918, Fritts 1965, Zweifel et al. 2021). In addition to meteorological drivers, the radial growth rate of boreal and temperate trees follows the intra-annual variation of the photoperiod (Huang et al. 2020) and may be sensitive to direct solar irradiance (Kašpar et al. 2024).

Intra-annual dynamics of radial stem growth can be monitored through xylogenesis sampling or using automated dendrometers (Steppe et al. 2015). Ideally, both approaches are combined to obtain a compelling picture of growth phenology and kinetics, taking into account the specific limitations of each method (Cruz-García et al. 2019, Francon et al. 2024). Xylogenesis monitoring involves collecting wood microcores to prepare microscopic sections of the cambial zone and the developing xylem. This approach allows for the direct counting of cells forming a tree ring, often distinguishing between different ontogenetic stages (Rathgeber et al. 2016). Since the sampling frequency typically varies between 1 and 2 weeks, the resolution of xylogenesis data is insufficient to provide insights into sub-daily growth variations and their meteorological drivers. This limitation can be overcome by using automated dendrometers, i.e., electronic devices mounted on tree stems that continuously measure their radius, diameter or girth (MacDougal 1921, Drew and Downes 2009). However, the signal of irreversible wood formation recorded by dendrometers is partly masked by changes in stem water content and hygroscopic effects of the bark, which lead to stem shrinkage and swelling (Chan et al. 2016, Delapierre et al. 2023). Although these reversible oscillations can be statistically filtered out (Aryal et al. 2020, Knüsel et al. 2021), dendrometer records are only an indirect proxy for xylogenesis, particularly for cell production and enlargement rather than wall lignification (Cuny et al. 2015). Validating dendrometer records with direct xylogenesis observations is necessary to understand seasonal offsets between stem radius increment and xylem differentiation (Cruz-García et al. 2019, Silvestro et al. 2023).

In this study, we evaluate intra-annual and sub-daily growth dynamics and their meteorological drivers for the cold-dry treeline of *Pinus sylvestris* L. in Northern Sweden beyond the Arctic Circle. We monitored (bi-)weekly xylogenesis for 2 years and measured hourly stem circumference using automated dendrometers. To assess the direct and lagged effects of meteorological variables on radial growth, we applied correlation analyses between meteorological variables and growth series, considering both immediate (i.e., concurrent meteorological and growth series) and legacy effects (i.e., meteorological series preceding growth series by 1 to 192 h). Moreover, we applied a (cross-)wavelet power transformation analysis, i.e., a mathematical convolution of the dendrometer and meteorological series (Qian et al. 2007), which is a proven tool to test for the presence of periodic, non-stationary oscillations in time series, such as daily, seasonal and multiannual cycles in tree growth (Oberhuber et al. 2015, Perone et al. 2016, Muraja et al. 2025). We hypothesized that stem radius variation recorded by dendrometers, which is driven particularly by cell expansion (Cuny et al. 2015), would show frequency-dependent responses to environmental variables. Specifically, we assumed that the stem radius increment at the seasonal scale would follow temperature and photoperiod, resulting in peak increases during the warm summer. However, we expected stem radius increment to be driven by moisture availability at the sub-daily scale, resulting in peak increases during the cool, humid part of the day with the sun close to the horizon. Accordingly, we expected to find a non-linear relationship between stem radius increments and immediate meteorological conditions, with growth increasing with temperatures only when air humidity remains sufficiently high.

## Materials and methods

### Study site and study species

The monitoring of stem growth and wood formation was conducted near Abisko, Northern Sweden (N 68.350°, E 18.805°; Figure S1 and Figure S2 available as Supplementary Data at *Tree Physiology* Online). The area represents a transition zone between boreal forest and treeless tundra with scattered mosaics of forest patches on dry ridges and with peatland vegetation in wet valleys and flat landscapes. Sparse forest stands are mainly composed of solitary *P. sylvestris* with shrubby habits of *Betula pubescens* ssp. *czerepanovii* (mountain birch) in the understory. The climate is a cold subarctic with low precipitation sums due to a rain shadow from the Scandinavian Mountains. The mean annual temperature is 0.6 °C and total annual precipitation is 349 mm (Swedish Meteorological and Hydrological Institute; station Abisko 2002–2020). Mean daily temperatures peak in July (12.9 °C) and the lowest mean daily temperatures occur during January (−10.5 °C). Summer is the rainiest season of the year, while spring is the driest. The polar day lasts for 53 days from 27 May to 18 July.

*Pinus sylvestris* is an evergreen conifer with a wide ecological niche, reaching its northern distribution limit in Northern Fennoscandia (Caudullo et al. 2017). It is a light-demanding species well adapted to sustain climatic extremes, including summer drought stress at dry sites and harsh cold winters in the northern boreal forests (Ellenberg and Leuschner 2010, Bose et al. 2020). With ongoing climate warming, Scandinavian populations of *P. sylvestris* are expected to accelerate growth toward the end of the 21st century in response to alleviating the cold limitation of their cambial activity (Martinez del Castillo et al. 2024).

### Monitoring of hourly stem size variation and meteorological variables

We installed automatic band dendrometers of the type DRL26C (EMS Brno, Czech Republic) on three dominant and healthy trees (mean diameter at breast height was 31 cm) in late March 2023 before the start of the growing season. Dendrometers were installed on straight stems at a height of 1.3 m above the ground to measure the hourly variation of their circumference. Outer layers of dead bark were gently peeled before the installation of the band to reduce hygroscopic and freezing effects on the dendrometer readings (Zweifel and Häsler 2000, Delapierre et al. 2023). The devices operated without interruption for 2 years, and the measured data were downloaded in April 2025.

Dendrometer data were visually inspected and statistically corrected for the rare presence of artificial jumps (Aryal et al. 2020). Measurements of stem circumference changes were converted to stem radius changes assuming a circular shape of the cross-section (i.e., dividing the circumference by 2π). We used the ‘zero-growth’ approach to filter out reversible shrinking and swelling, driven mainly by changing amounts and the state of water inside the tree, from the proxy for irreversible stem growth (Zweifel et al. 2016). Accordingly, differences in instantaneous stem radius exceeding the previous maximum were ascribed to irreversible increment of the stem size (stem growth; GRO), while differences below the previous maximum were filtered out as reversible stem variation driven by the tree water deficit. Values of GRO for April 2023, i.e., the first month after the installation of dendrometers, were excluded from further analysis due to possible noise related to the adjustment of the band and sensor on the bark surface. In spring 2024, we noticed a significant, up to 4 weeks' delay of the first non-zero GRO compared with our direct monitoring of cambial activity (see below section Monitoring of xylogenesis). Due to strong winter stem shrinkage, stem size had not reached the previous maximum although cambial activity was already in progress. As previous reports from the Subarctic indicated suboptimal performance of band dendrometers (Francon et al. 2024), we mathematically adjusted the zero-growth approach in 2024 to improve the agreement of the GRO series with xylogenesis data. To do this, we replaced the value of the previous stem maximum on 15 May 2024 at 01:00 h with the stem size measured at midnight of the previous day for each dendrometer before running the zero-growth approach. Accordingly, we simulated non-zero GRO during the second half of May and early June 2024, which otherwise would be masked by stem shrinkage. Rare non-zero GRO values associated with stem swelling due to sudden air warming in December 2024 (Figure S3 available as Supplementary Data at *Tree Physiology* Online) were excluded from further analysis.

Additionally, we installed sensors that continuously recorded air and soil microclimate at an hourly temporal resolution at the site during the same period (March 2023 to April 2025). Specifically, two TEROS 21 sensors (METER Group, USA) measuring soil water potential and soil temperature were buried at a depth of 25 cm below the soil surface. Both devices recorded similar values for temperature and water potential, mainly during the warm part of the year, and we averaged their data for the later steps of the analysis. Moreover, we used a Minikin TH2/R (EMS Brno, Czech Republic) placed in a radiation shield to measure air temperature and relative air humidity at a height of 2 m above the ground below the tree canopy. Hourly air temperature and air humidity series were used to calculate the hourly vapor pressure deficit (VPD; Duursma 2015).

### Monitoring of xylogenesis

We sampled microcores from seven dominant and healthy trees (mean diameter at breast height was 37 cm), including the three trees equipped with dendrometers, to determine the timing and rates of xylem cell production. The sampling was performed weekly or bi-weekly between 24 April 2023 and 20 October 2023 and between 13 May 2024 and 24 September 2024. We punched the Trephor tool (Rossi et al. 2006*a*) into the stem using a plastic hammer and extracted a narrow cylinder of bark, phloem, cambium and xylem. The sample was immediately stored in a fixative to preserve xylem cells in their respective developmental stage. Laboratory processing of microsamples followed standard protocols for producing permanent microscopic slides of plant tissues (Gärtner and Schweingruber 2013). Specifically, each sample was dehydrated using ethanol, embedded into paraffin and cut using a rotary microtome to cross-sections with a thickness of 15 μm. Next, cross-sections were double-stained using Safranin and Astrablue and mounted on permanent microscopic slides with Canada balsam. We observed each cross-section using an optical microscope in transmitted and polarized light with up to ×400 magnification and counted cells along three radial tracheid files of the forming ring. Moreover, we assessed the developmental stage of each cell according to the shape and size of the lumen, wall thickness, progress of wall lignification and presence or absence of protoplast (Rathgeber et al. 2016). Accordingly, we distinguished between cambial cells (CZ), xylem cells in the phase of lumen enlargement (EN), xylem cells in the phase of wall thickening and lignification (WT), and mature xylem cells without protoplast (MC).

We also counted the cell numbers in the previous ring along three radial files and subsequently used them to standardize the variation in growth rate around the stem circumference. To do this, we multiplied the cell numbers of each stage (CZ, EN, WT, MC) in each sample by the mean number of previous year’s cells across all samples divided by the number of previous year’s cells in that sample (Rossi et al. 2003). Standardized numbers of cells in the three independent radial files were averaged for each tree and sample. These weekly or bi-weekly data of cell counts were interpolated for each day of the year (DOY) and each tree by fitting the Gompertz function to cumulative numbers of xylem cells in any phase of development (EN + WT + MC; Rossi et al. 2003). We calculated daily rates of cell production as the first differences of Gompertz functions for each tree. In addition, we determined phenological dates of wood formation as the first and the last days with the presence of cells in a given developmental phase in at least two of the three radial tracheid files (Rathgeber et al. 2018). Accordingly, we calculated the dates for the onset of EN, WT and MC phases and for the cessation of EN and WT phases for each tree and year.

### Growth patterns from dendrometer and xylogenesis records

#### Visualization of sub-daily patterns from dendrometers

We averaged GRO for each hour of the day (Zweifel et al. 2021) and for intervals of solar altitude in steps of 5° during the main climatological seasons (winter from December to February; spring from March to May; summer from June to August; autumn from September to November). We calculated the hourly solar altitude as the vertical angle between the sun and the horizon at the coordinates of our site (Kelley 2018). For each hour of the day and solar altitude step, we also calculated 95% confidence intervals for mean GRO and compared them with the seasonal mean GRO to identify hours of the day and solar positions with significantly below- or above-average GRO rates.

#### Visualization of intra-annual growth patterns from xylogenesis

We plotted the number of cells in individual stages of development observed on xylogenesis samples during each sampling date to characterize their intra-annual patterns over the year. We also plotted the first differences of Gompertz models fitted to the cumulative numbers of cells as a proxy for the daily rate of cell production. To statistically evaluate the coherence between the xylogenesis and dendrometer growth series of three trees, we calculated the mean GRO rates and the mean number of EN and WT cells for each period between consecutive xylogenesis sampling dates. We correlated the averaged GRO series with the numbers of EN and WT cells to assess how GRO recorded by dendrometers is controlled by cell enlargement and wall-thickening.

### Environmental drivers of radial growth

#### Environmental drivers of sub-daily growth from dendrometers

We carried out Pearson correlations between hourly GRO series and the immediate hourly meteorological variables (air and soil temperature and humidity). To identify seasonal shifts in environmental controls of stem radius variation (Kocher et al. 2012, Debel et al. 2024, Bose et al. 2025), these correlations were calculated in biweekly intervals of 14 consecutive calendar days. To quantify lagged effects of meteorological variables on radial growth (van der Maaten et al. 2018), we correlated hourly GRO with the respective meteorological variables recorded 1 to 192 h (8 days) before. A partial version of the Pearson correlation coefficient was used to remove the potential effect of daily cycles on lagged correlations (Simms et al. 2022), i.e., we first calculated residuals of the linear regressions of GRO and each meteorological variable on the solar altitude, and then calculated the correlation of these residuals between GRO and meteorological variables. Different approaches for removing daily cycles, such as detrending with the sine-cosine function or 24-h moving average, yielded similar patterns of correlations.

To identify the range of optimal meteorological conditions for the growth of *P. sylvestris* at our site, we converted the continuous meteorological variables and solar altitude into categorical intervals and calculated mean values of GRO from all timesteps with conditions within each interval (Güney et al. 2020). The stepsize of the non-overlapping intervals was determined for each environmental variable based on its range and histogram as follows: 4 °C (air temperature), 2 °C (soil temperature), 25 kPa (soil water potential), 0.2 kPa (VPD) and 5° (solar altitude). Based on differences in mean GRO between intervals, we determined the range of optimal meteorological conditions during which the majority of the annual tree ring was formed, and the GRO rate peaked. To assess shifts in optimal intervals of meteorological conditions during the day, we separately calculated GRO means for these intervals if the sun was below the horizon (solar altitude <0°), close to the horizon (0° < solar altitude < 15°) and high above the horizon (solar altitude >15°). Differences in GRO among the three solar altitude intervals were tested with analysis of variance (ANOVA).

#### Environmental drivers of intra-annual growth in xylogenesis data

To statistically evaluate the relationship between bi-weekly xylogenesis and hourly meteorological data, we employed the approach introduced by Cuny et al. (2013). We used the first derivatives of the Gompertz functions to estimate the timing of enlargement and wall-thickening for each cell. Next, we calculated mean values of meteorological variables recorded during both phases of xylogenesis for each cell and compared them over the growing season (Cuny and Rathgeber 2016).

#### Linear mixed-effect models

We used generalized linear mixed-effects models to quantify the linear covariance of growth series derived from both dendrometer and xylogenesis records with meteorological variables, accounting for different sources of random variation. Three types of growth series were tested as response variables in the models, namely GRO rates derived from dendrometers, GRO occurrence (a binary variable indicating whether GRO rate exceeded zero; Etzold et al. 2022) and first differences of cell numbers derived from Gompertz models (xylogenesis). For the model on GRO rates, only timesteps with non-zero growth were used as input. Recorded and derived meteorological variables were used as fixed predictors, including VPD, soil water potential and the interaction between air and soil temperature (due to their collinearity). We performed the analysis at hourly (only dendrometer data), daily and weekly temporal resolutions (dendrometer and xylogenesis data). Random intercepts were modeled between trees (included in all models), days of year (included in daily and hourly models) and hours of day (only in the hourly model).

### Wavelet analysis

We carried out a wavelet power transformation to test for the presence of periodic oscillations in the GRO series, to rank the importance of oscillations with different frequencies and to quantify their statistical significance (Oberhuber et al. 2015, Chan et al. 2018, Tumajer et al. 2023). Wavelet power transformation is a mathematical convolution testing for the agreement between harmonic oscillations in time series and ‘wavelets’, i.e., artificial wave-like signals with known frequency and position in time (Qian et al. 2007). This approach represents an effective tool for analyzing series non-stationarity in frequency and amplitude over finite time periods, which was previously used for studying periodic oscillations in tree-ring chronologies (Perone et al. 2016, Muraja et al. 2025) and dendrometer growth series (Oberhuber et al. 2015). Accordingly, this approach is well suited to unravel whether stem radius shows seasonal and daily cycles in addition to the long-term increasing growth trend. We generated 254 wavelets of the Morlet mother type (Roesch and Schmidbauer 2018) with a period between 8 and 12,854 h (≈1.5 years) and shifted them in steps of 1 h from the first to the last timestep of the dendrometer series (from 1 May 2023 00:00 h to 16 April 2025 00:00 h). At each position, we calculated the ‘wavelet power’ and its significance to quantify the agreement of local variation in GRO series with each wavelet. We plotted a matrix of standardized wavelet power decomposing harmonic oscillations in stem dimensions into a time-frequency domain. We used the matrix to identify seasons of the year and frequencies of oscillations with significant (*P* < 0.01) wavelet powers, evidencing systematic cycles in our GRO series. The wavelet power transformation was performed on the site level for the mean GRO series across all three trees equipped with dendrometers.

Furthermore, we performed a cross-wavelet transformation to quantify the similarities of the wavelet power spectra of the mean GRO series with the spectra of the considered meteorological variables and solar altitude (Torrence and Compo 1998). Mathematically, the cross-wavelet transformation represents the covariance between wavelet power spectra of two series, i.e., the cross-wavelet power is highest if both series show high wavelet power for a given frequency in a given time. Similar to wavelet powers, we visualized cross-wavelet powers for each pair of dendrometer and environmental variables by plotting the matrix in the time-frequency domain. In addition to cross-wavelet power, we calculated a phase offset between both series to quantify how peaks in GRO lag behind peaks in environmental variables. We used absolute values of phase offset for individual frequencies with significant (*P* < 0.01) cross-wavelet power to assess whether GRO and environmental series are in-phase and their peaks occur in synchrony (|offset| = 0), in anti-phase with a peak of one series synchronized with a minimum of the other series (|offset| = π) or whether there is another systematic temporal shift between series peaks (0 < |offset| < π).

All processing of the data, statistical analyses and visualizations were performed in R 4.2.2. (R Core Team 2025) using packages ‘dendRoAnalyst’ (Aryal et al. 2020), ‘treenetproc’ (Knüsel et al. 2021), ‘CAVIAR’ (Rathgeber et al. 2018), ‘plantecophys’ (Duursma 2015), ‘oce’ (Kelley 2018), ‘WaveletComp’ (Roesch and Schmidbauer 2018), ‘lme4’ (Bates et al. 2015) and ‘ggplot2’ (Wickham 2009).

## Results

### Intra-annual and sub-daily growth patterns

**Figure 1:**
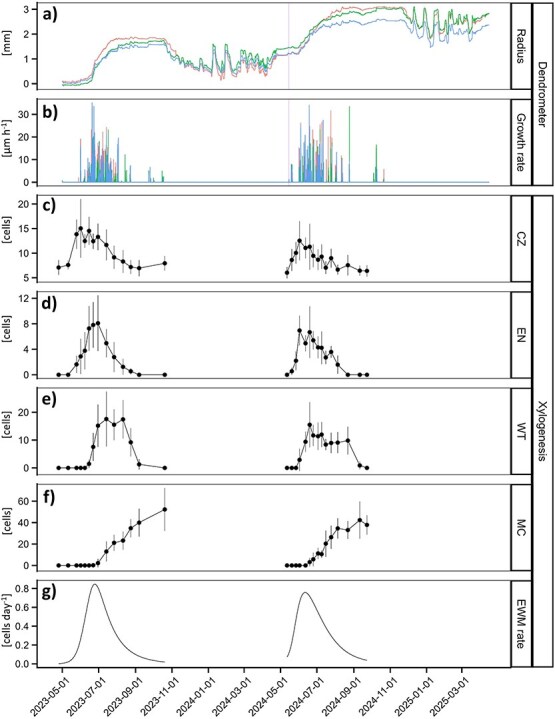
Dendrometer and xylogenesis records of intra-annual radial growth and cell increment. Dendrometer records (a, b): hourly-resolved data for stem radius increment and radial growth rate for three trees (colors). The purple vertical line indicates 15 May 2024, when the zero-growth approach for calculating the growth rate was adjusted for the 2024 growing season. Cell numbers from xylogenesis data (c–f): numbers of cells in different phases of differentiation counted on xylogenesis samples that were collected weekly or biweekly from seven trees (points with error bars representing mean ± standard deviation). CZ = cambial cells; EN = enlarging cells; WT = wall-thickening cells; MC = mature cells; daily cell number increment from xylogenesis data (g): mean of day-to-day differences of Gompertz models fitted separately to each of seven trees. EWM rate = daily increment in the number of enlarging, wall-thickening and mature cells.

The mean annual radial increment recorded by dendrometers and the mean number of produced mature cells were 1.69/1.64 mm and 52/42 in 2023 and 2024, respectively (Figure 1). Non-zero GRO rates derived from dendrometers and the enlargement phase determined from xylogenesis monitoring (occurrence of EN cells) roughly overlapped, mainly between the end of May (mean DOY of first EN cells was 144/140 in 2023/2024) and early-mid August (last EN cells on DOY 236/224; Figure 1 and Figure S4 available as Supplementary Data at *Tree Physiology* Online). This period was followed by a phase with irregular occurrence of marginal non-zero GRO in September and October. The WT phase lasted from the first half of June (DOY 166/154) to mid-September (DOY 255/252). Accordingly, the duration of the EN (92/84 days) and WT phases (89/98 days) was comparably long. The rate of cell production per day, estimated as a first difference of Gompertz functions fitted to xylogenesis data, peaked in June (DOY 176/162). Mean GRO between two consecutive xylogenesis sampling dates was significantly correlated with the mean number of EN cells during this interval for all trees, but the correlations between GRO and WT cells were mostly non-significant (Figure S5 available as Supplementary Data at *Tree Physiology* Online).

**Figure 2:**
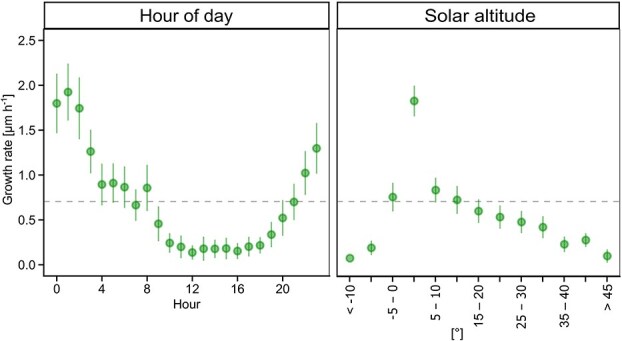
Mean radial growth rate of the three dendrometer trees per hour of the day (left) and five-degree intervals of solar altitude above the horizon (right) during the summer months (June–August). The points and error bars represent the mean and the 95% confidence intervals, respectively. The horizontal dashed line indicates the mean hourly growth rate during the summer (0.71 μm h^−1^). Growth is largest at 00:00–02:00 h and with the sun 0–5° above the horizon. For other seasons and separate charts for individual trees, see Figure S6 available as Supplementary Data at *Tree Physiology* Online.

Regular sub-daily variations of GRO rates occurred during the summer months (June–August) but were absent in other seasons due to low GRO values from September to May (Figure 2 and Figure S6 available as Supplementary Data at *Tree Physiology* Online). Mean GRO rates significantly exceeded the seasonal summer mean GRO from 22:00 to 03:00 h, with peak GRO rates around midnight (00:00–02:00 h). By contrast, GRO was significantly below-average from 09:00 to 19:00 with a minimum shortly after midday (14:00–15:00 h). Significantly above-average growth characterized the range of solar altitudes 0–5° and significantly below-average growth occurred when solar altitude was <−5° or >20°.

### Environmental drivers of intra-annual and sub-daily radial growth

**Figure 3:**
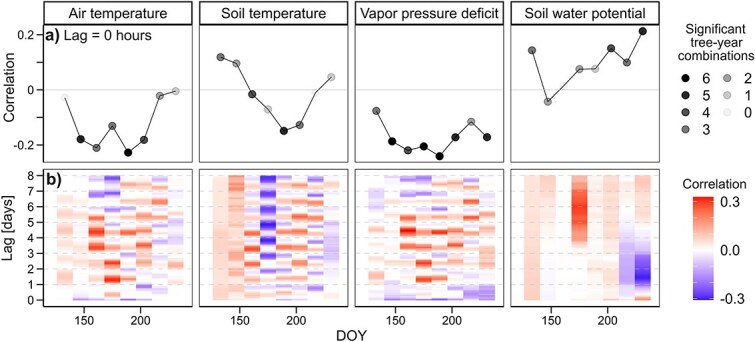
Partial correlation coefficients between hourly radial growth rates and immediate meteorological variables in hourly resolution for each biweekly period (a) and matrix of partial correlation coefficients between hourly radial growth rates and lagged meteorological variables in hourly resolution for each biweekly period (b). The effect of solar altitude on partial correlations between growth rates and meteorological variables was removed as a confounding variable. The y-axis in (b) indicates the considered lag period between the radial growth series and the meteorological variables, ranging from 1 h to 8 days (192 h). Correlations are plotted for the main part of the growing season (DOY 120–240) on the day of the year that is in the center of each biweekly period. Both immediate and lagged partial correlations were calculated separately for each independent combination of tree and year (six possible combinations in total) and averaged before plotting. The transparency of the points (a) and pixels (b) represents the number of tree-year combinations for which the given correlation is significant with *P* < 0.05.

Correlations between meteorological variables and GRO peaked between DOY 150 and 220 (i.e., June to July; Figure 3a) when GRO was largest. Hourly GRO rates were significantly negatively correlated with immediate air temperature and VPD in summer. Correlations with immediate soil water potential and soil temperature were weaker, less significant and switched between positive and negative throughout the growing season. Notably, correlations of the GRO series with lagged air temperature and VPD preceding growth for 36, 60 and 84 h were positive during peak summer around DOY 170 (Figure 3b).

The air temperature that each cell experienced during the EN and WT phases remained relatively stable (12–15 °C) over the growing season, except for the first and the last cells in the radial file (Figure S7 available as Supplementary Data at *Tree Physiology* Online). Mean VPD that cells experienced during their EN and WT phases declined over the year in 2023, but not in 2024. Notably, soil conditions were systematically cooler and wetter during the EN phase compared with the WT phase for most cells, except for the last cells before the end of the growing season.

Linear mixed-effects models explaining GRO rate and GRO occurrence confirmed a significant negative covariance with VPD and a weaker positive correlation with soil water potential and temperature at hourly and daily scales (Table S1 and Table S2 available as Supplementary Data at *Tree Physiology* Online). For daily cell production derived from xylogenesis monitoring, soil water potential was the only statistically significant predictor, positively affecting cell production. The VPD effect switched from negative to positive in models of cell production, GRO rate and GRO occurrence on the weekly scale.

### Optimal meteorological conditions for the growth of *P. sylvestris* at the Arctic treeline

**Figure 4:**
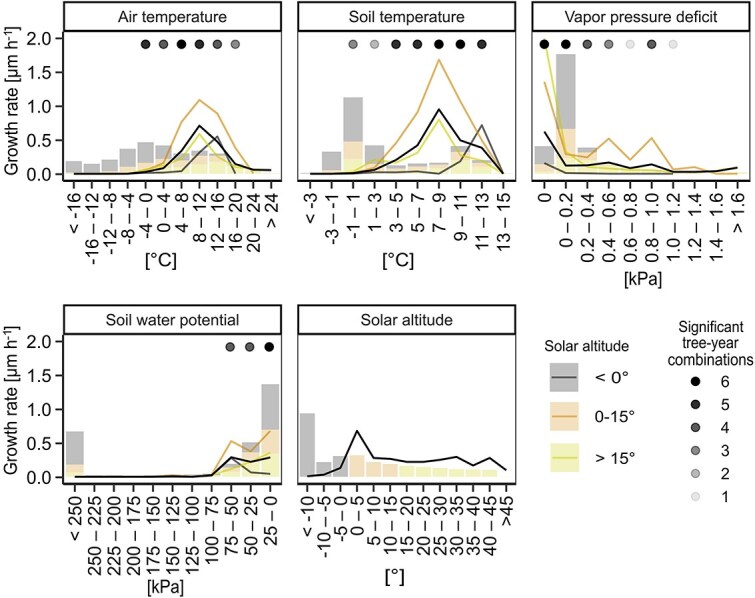
Mean radial growth rates for specific non-overlapping intervals of the environmental variables (lines) with the relative frequency of timesteps with environmental variables within the given interval (bars). Black lines show mean growth rates irrespective of the solar altitude. Gray, orange and yellow lines and bars show mean radial growth rate and relative frequency of timesteps for specific intervals of solar altitude (below horizon, near horizon, high above horizon). The transparency of the points at the top of each chart indicates the number of independent tree-year combinations (six possible combinations in total) for which ANOVA testing for differences in mean growth rates between three intervals of the solar altitude is significant with *P* < 0.05.

**Figure 5:**
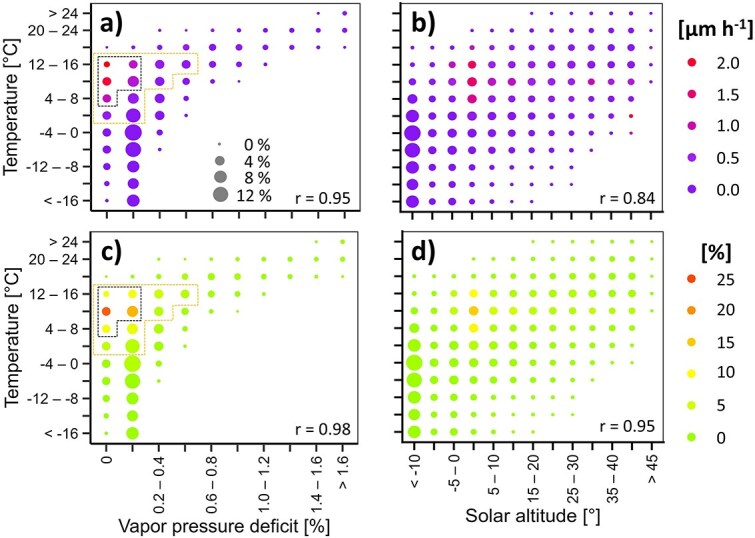
Scatterplots of air temperature and vapor pressure deficit (a, c), and air temperature and solar altitude above the horizon (b, d) for hourly timesteps. The color of the dot indicates the mean radial growth rate (a, b) and the relative proportion of the mean annual growth under the specific combination of environmental variables (c, d). The size of each dot is proportional to the relative frequency of timesteps with the given combination of environmental variables between May 2023 and April 2025 (the scale at the bottom of panel (a) applies to all panels). The area highlighted with dashed black lines shows the critical interval of the climatic optimum with the highest mean growth rates, which contributed 68% to the total stem radial increment between May 2023 and April 2025. Dashed brown lines delimit climatic space of suboptimal growth, which contributed another 22% to the annual stem increment. Remaining part of climatic space outside dashed brown lines contributed 10% to the annual stem increment. r values in the bottom-right corner of each panel represent the mean Mantel statistics that test the similarity of the scatterplots between individual trees. All Mantel tests were highly significant (*P* < 0.001), indicating low variability between trees.

The highest mean GRO occurred at intervals of 8–12 °C air and 7–9 °C soil temperature, and at low values of VPD and soil water potential close to 0 kPa, i.e., the dew point and fully water-saturated soils (Figure 4). Notably, there was a sharp decrease in mean GRO by over 80% when air humidity was reduced from dew point to slightly drier air (0 < VPD < 0.2 kPa). If the sun was below the horizon, the GRO was less dependent on VPD, but the growth peak shifted to higher temperatures. By contrast, the importance of VPD as a driver of GRO rate increased during the day with solar altitude above the horizon. ANOVA pointed out significant differences in mean GRO between solar altitudes, mainly for intervals with high mean growth rates. Consequently, optimal conditions for growth of *P. sylvestris* at our site, i.e., combinations of meteorological variables with the highest mean GRO, were reached at (i) an air temperature of 4–16 °C and a VPD = 0 kPa (mean GRO within this interval was 1.5 μm h^−1^), and (ii) an air temperature of 8–16 °C and 0 kPa < VPD < 0.2 kPa (0.7 μm h^−1^). The mean GRO within both intervals of this optimum together was 1.1 μm h^−1^ (Figure 5a and b), and sum of GRO within this optimum accounted for 68% of the mean annual sum of GRO (Figure 5c and d).

### Wavelet analysis

**Figure 6:**
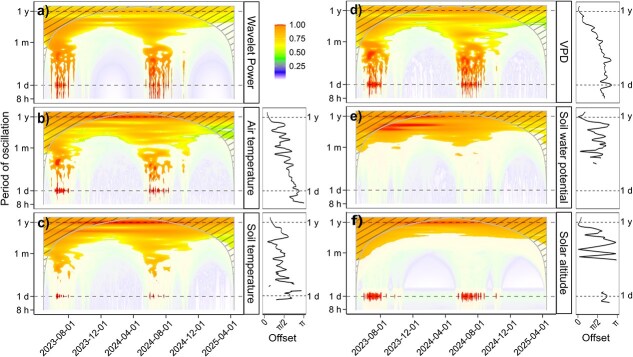
Matrixes showing the wavelet power spectra of the radial growth rate (a) and the cross-wavelet power spectra for its covariance with meteorological variables and solar altitude (b–f). X-axes indicate 1-h timesteps and Y-axes indicate the period of wavelet oscillation (h = hour, d = day, m = synodic month ≈ 29.53 days, y = year). The color gradient represents the standardized (0–1) power of the wavelet, with red and blue colors indicating oscillations with the highest and lowest power, respectively. Pixels with an estimated *P* > 0.01, i.e., non-significant wavelet or cross-wavelet power, are semitransparent. Hatched polygons highlight the cone of influence with significant edge effects due to limited replication. The line charts to the right of the cross-wavelet matrixes (b–f) show the mean absolute value of the phase offset between the environmental variables and the radial growth rate. Only periods and timesteps with significant (*P* < 0.01) cross-wavelet power were used for the calculation of the offset. X-axes indicate the offset, with 0 representing in-phase oscillations (i.e., the peaks of both series are synchronized) and π representing anti-phase oscillations (i.e., the peaks of one series occur during minima of the other series). VPD = vapor pressure deficit.

The wavelet power transformation revealed significant oscillations in the GRO series with periods of 24 h and 1 year (Figure 6a). The daily oscillation was significant only during the growing season, approximately between June and August. Its cross-wavelet power peaked under covariance with solar altitude and VPD (Figure 6b–f). A weaker, but significant daily cross-wavelet power was found with air temperature. The mean temporal offset between the daily cycles in GRO and environmental variables varied between ¾π and π, indicating that the GRO peaks were shifted by 9–12 h from the daily peaks of temperature, VPD and solar altitude. The offset of the annual cycle in the cross-wavelet power spectra was close to 0, indicating coherence between oscillations in GRO and environmental variables. This suggests that the annual GRO peaks occur in synchrony with annual maxima of air and soil temperature, VPD, soil moisture and solar altitude.

## Discussion

By monitoring radial stem increment and wood formation at bi-weekly (xylogenesis monitoring) to sub-daily (dendrometer records) resolution, we demonstrate that intra-annual growth in a cold-dry treeline ecotone beyond the Arctic Circle (i) consisted of overlapping annual and daily oscillations and (ii) exhibited frequency-dependent sensitivity to meteorological variables. Radial growth accelerated during the warm season, resulting in peak cambial activity and radial stem increment during the summer months. However, within the summer season, the radial stem increment systematically peaked during the cooler, more humid parts of the polar day shortly after midnight when the sun was close to the horizon. Consequently, mixed-effects models and cross-wavelet power transformation jointly confirmed that the influence of meteorological factors on growth depended on the temporal resolution of the analysis. Furthermore, immediate and lagged effects of temperature and humidity on hourly tree growth showed inverted patterns, suggesting that the growth rate peaked during humid, cool summer nights following a sequence of relatively warm, dry days. Below, we discuss the frequency-dependent sensitivity of stem radial growth and the immediate and lagged meteorological effects together with the implications of our results for future growth of Arctic treeline ecotones under a warming climate.

### Annual and daily cycles in dendrometer and xylogenesis records

Our results revealed a short growing season from late May to August, during which we observed the production of new cells and their progression into the expansion phase, as well the majority of non-zero growth rates recorded by dendrometers (Figure 1). The mean duration of ~3 months for both the enlarging and wall-thickening phases of xylogenesis is in line with the global minimum expected at treeline sites (Körner and Hoch 2023) and with the short window of temperature sensitivity typical for subarctic tree-ring width chronologies (Andreu-Hayles et al. 2020). Accordingly, the cross-wavelet transformation indicated significant synchronization between the annual cycle of radial growth recorded by the dendrometers and air and soil temperature, humidity and solar altitude. Furthermore, the linear mixed-effects models in weekly resolution highlighted a positive influence of VPD on the rate of xylogenesis and radial growth (Zhou et al. 2023). In other words, on an annual scale, peak growth rates occurred during the warmest and driest (in terms of air humidity) part of the year with the longest daylength. This is in agreement with previous studies monitoring the annual growth cycle in cold environments (Rossi et al. 2006*b*, Treml et al. 2015, Buttò et al. 2019, Obojes et al. 2022, Francon et al. 2024). Furthermore, this is in line with boreal forest increments, derived from tree-ring series (Esper et al. 2016) or repeated forest inventories (Pretzsch et al. 2023), responding positively to seasonal temperature when analyzed at annual or multiannual resolution.

In contrast, on a sub-daily scale, the stem radial increment consistently increased as solar altitude and thus temperature decreased, reaching its peak around midnight when the sun was near the horizon and air humidity was highest (Figure 2). Consequently, our cross-wavelet power analysis pointed out up to 12-h offset of daily growth peaks from midday maxima of temperatures and VPD. In line with this, Pearson correlations and linear mixed-effects models revealed a negative covariance of hourly growth rate with immediate VPD. Nocturnal radial growth of tree stems was previously reported from various biomes (Boulouf Lugo et al. 2012, King et al. 2013, Ziaco and Biondi 2018, Güney et al. 2020) and was associated with reduced transpiration and increased turgor pressure in cambial zone after sunset (Steppe et al. 2015). We show for the first time that these daily cycles in stem radial growth exist beyond the Arctic Circle and persist during the polar day. Notably, while stem growth in temperate climates typically peaks before sunrise after overnight turgor replenishment (02:00–06:00 h; Zweifel et al. 2021), maximum summer growth rates at our Arctic treeline site were shifted closer to midnight (00:00–02:00 h). This illustrates a tight coupling of radial growth to daily cycles of meteorological variables regardless of whether the sun stays constantly above the horizon during the polar day.

### Meteorological drivers of radial growth

Hourly stem growth rates derived from dendrometer records showed systematic relationships to meteorological variables with a peak at air temperatures between 8–12 °C and a decline toward both cooler and warmer conditions. Previous studies have shown that positive tree growth responses to increasing temperatures fade out or invert after exceeding a threshold of 13–16 °C (Carrer et al. 1998, Sidor et al. 2015, Piovesan et al. 2023). This range corresponds to the upper edge of our window of optimal growth (Figure 4) and to the mean air temperature experienced by most cells during the enlargement and wall-thickening phases throughout the growing season (Figure S7 available as Supplementary Data at *Tree Physiology* Online). The mean hourly growth rate consistently decreased with increasing VPD and decreasing soil water potential, particularly if the sun was high above the horizon. Specifically, the mean growth rate dramatically dropped in response to increasing VPD from 0.63 μm h^−1^ during dew point to 0.12 μm h^−1^ in slightly drier air (0 kPa < VPD < 0.2 kPa). Moreover, we observed significant differences in growth rates between independent intervals of climatic optima combining air temperature and VPD: During the dew point, tree growth is very effective across a wide range of temperatures (i.e., mean growth rate is 1.5 μm h^−1^ across the range of 4–16 °C air temperature) but it declines significantly under slightly drier and warmer air (0.7 μm h^−1^ at 8–16 °C; Figure 5a). This suggests that, although the position of the treeline is biogeographically constrained by low temperatures (Körner and Hoch 2023), the sub-daily radial stem increment at our Arctic treeline site becomes primarily limited by immediate air humidity rather than temperature once a certain temperature threshold of cambial activity is exceeded.

Correlations between instantaneous meteorological variables and hourly growth series showed negative effects of temperature and VPD on radial growth, mainly during summer. However, responses of radial growth to lagged meteorological variables were often inverted to their immediate effects. For instance, peak-summer radial growth rates were positively correlated with air temperature and VPD at 36, 60 and 84 h before the specific radial growth timestep. Considering the nocturnal occurrence of radial growth (Figure 2; Zweifel et al. 2021), the growth statistically benefited from a sequence of relatively warm and dry days followed by cooler and wetter nights. This might partly be due to the persistence of the daily oscillations in the residuals of the meteorological and growth series (Simms et al. 2022), but may also reflect the lagged effects of environmental variables on tree growth. For instance, high temperature in a preceding period might act as an important pre-conditioning factor stimulating wood formation (van der Maaten et al. 2018), mainly at the treeline with substantial thermal demand for cell production (Körner 2003).

### Comparison of xylogenesis and dendrometer monitoring

Our experimental setup offered a unique opportunity to compare dendrometer records with xylogenesis monitoring, as both methods were applied to the same trees for a subset (*n* = 3), and the remaining xylogenesis trees (*n* = 4) were growing in very close proximity. Although we obtained dendrometer data only for three trees, their growth series were coherent (Figure 1a), their responses to meteorological conditions were similar (Mantel tests associated with Figure 5) and linear-mixed effects models confirmed lower between-tree variation in stem increment compared with the variation over time (Table S1 available as Supplementary Data at *Tree Physiology* Online). This suggests that three dendrometers were sufficient to capture the local pattern of frequency-dependent oscillations in the stem size and their meteorological drivers.

Both dendrometers and xylogenesis monitoring proved their strengths and weaknesses for the intra-annual growth monitoring. Although dendrometers provide sub-daily data, they represent only an indirect proxy for the overall stem size variation. By contrast, xylogenesis monitoring enables a detailed distinction between phases of cambial division, cell expansion, and secondary wall deposition and lignification (Rathgeber et al. 2016) albeit in coarser temporal resolution. We confirmed that the radial growth rates recorded by dendrometers aligned well with the observed intra-annual pattern of enlarging cells, though not wall-thickening cells (Figure 1 and Figures S4 and S5 available as Supplementary Data at *Tree Physiology* Online). Dendrometers primarily measure the production of new cells in the cambial zone and their subsequent enlargement, but they do not reliably reflect cell wall lignification. Therefore, we emphasize the limited applicability of dendrometers for studies on the intra-annual carbon cycle in forest ecosystems, which should rather be addressed using xylogenesis monitoring or quantitative wood anatomy (Cuny et al. 2015, Krejza et al. 2022).

Considering growth onset in spring, we would have found a considerable offset in the timing of cell enlargement and the initial spring growth recorded by dendrometers had we not adjusted the zero-growth approach in May 2024. In the three trees with concurrent xylogenesis and dendrometer monitoring, we observed ongoing xylogenesis and the presence of new enlarging, wall-thickening and even mature cells before the stem radius reached the previous autumn maximum. This may have been due to the tendency of band dendrometers to obscure slow growth in early spring through the reversible shrinking and swelling of the bark (Francon et al. 2024) or the occurrence of cambial activity during periods of stem shrinkage not accounted for by the zero-growth approach (Zweifel et al. 2016). The latter suggests that trees in the cold Arctic treeline ecotone might be able to generate positive turgor in the cambial zone in the spring before reaching the previous maximum stem size. Interestingly, the opposite offset with xylogenesis data lagging behind dendrometers was observed for *P. sylvestris* in southern Finland (Chan et al. 2016). Overall, our and previous observations highlight the need for caution when simulating spring growth resumption using band dendrometers only, unless validated by direct monitoring of xylogenesis (Cruz-García et al. 2019, Adikurnia and Rathgeber 2025). We recommend that future studies test for potential diurnal lags in band dendrometers by comparing their measurements with other sub-daily growth series, e.g., records from more sensitive point dendrometers (Francon et al. 2024).

## Conclusions

Our results elucidate the frequency-dependent climate sensitivity of Arctic treeline trees with opposite responses of radial growth to environmental variables at annual and daily temporal scales: On the annual scale, tree growth peaked during the warmest season with the sun high above the horizon. On the daily scale, growth was favored by relatively cool and humid conditions around midnight. Therefore, we confirm the occurrence of diel cycles in stem radial growth, which were previously observed in various biomes in low and middle latitudes (Zweifel et al. 2021, Zhou et al. 2023, Luse Belanganayi et al. 2024), also beyond the Arctic Circle and during the polar day. Furthermore, tree growth in the summer was positively correlated with lagged temperature and VPD, while the immediate effects of these meteorological variables on tree growth were negative. This suggests that growth at the Arctic treeline might benefit from moist, cool nights within a relatively dry, warm summer season. We hypothesize—to be tested in the future—that the negative sensitivity of the nocturnal growth to immediate temperature and VPD may contribute to the weak response of some Arctic tree-ring width chronologies to recent warming (Briffa et al. 1998, D’Arrigo et al. 2008) and the emergence of moisture signals in cold-sensitive chronologies (Mirabel et al. 2023, including our site, Figure S8 available as Supplementary Data at *Tree Physiology* Online). We therefore advocate considering the frequency-dependent climate sensitivity of boreal trees and the inversion in their response to immediate and lagged meteorological effects when preparing growth projections under future climate change. To explore how landscape variability and soil conditions influence tree growth across Arctic treeline ecotones, we recommend tree growth monitoring at sites along environmental gradients, including soil water holding capacity, soil types, bedrock, slope orientation and permafrost thickness.

## Supplementary Material

Abisko_dendrometers_SM_revision2_tpag068

## Data Availability

Data supporting this study are available through Zenodo: https://doi.org/10.5281/zenodo.20022181.
