# Supplementary material for: Frequency-dependent climate sensitivity of sub-daily radial growth of tree stems at the dry Arctic treeline
Source: Tree Physiol. 2026 May 19;46(7):tpag068. doi: 10.1093/treephys/tpag068 (PMC13330539; doi:10.1093/treephys/tpag068)
Supplement: Abisko_dendrometers_SM_revision2_tpag068 [file abisko_dendrometers_sm_revision2_tpag068.docx]

# Frequency-dependent climate sensitivity of sub-daily radial growth of tree stems at the dry Arctic treeline

# *Jan Tumajer^1*^,* *Håkan Grudd^2^, Hana Kuželová^1^, Jelena Lange^1,3^, Václav Treml^1^*

1. *Department of Physical Geography and Geoecology, Faculty of Science, Charles University, Albertov 6, 12843 Prague, Czech Republic*
2. *Swedish Polar Research Secretariat, Abisko Scientific Research Station, Vetenskapens Väg 38, 981 07 Abisko, Sweden*
3. *Institute of Botany and Landscape Ecology, University of Greifswald, Soldmannstraße 15, 17487 Greifswald, Germany*

** -* [*tumajerj@natur.cuni.cz*](mailto:tumajerj@natur.cuni.cz)

**Supplementary material**

**
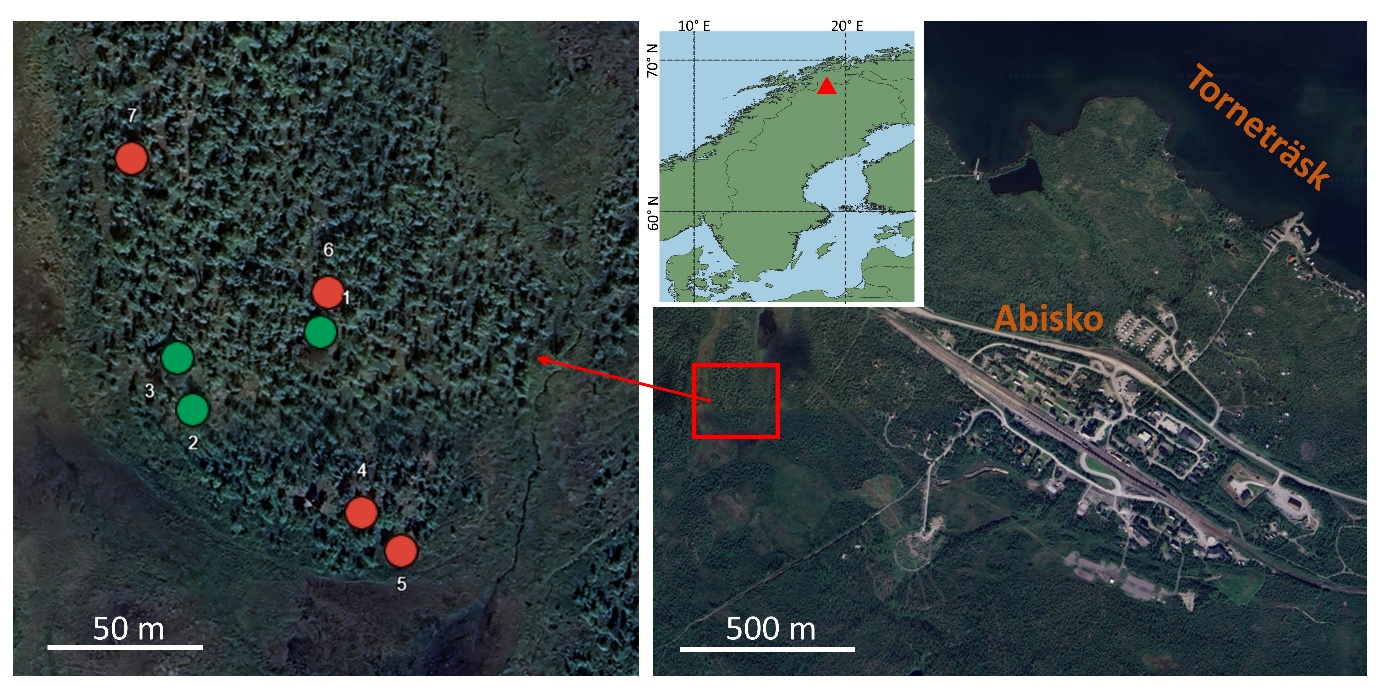
**

**Figure S1**: Study area in Abisko, Northern Sweden. Red points represent positions of trees with xylogenesis monitoring, green points are trees with simultaneous xylogenesis monitoring and dendrometers. The source of the orthophoto is Google Earth.

**
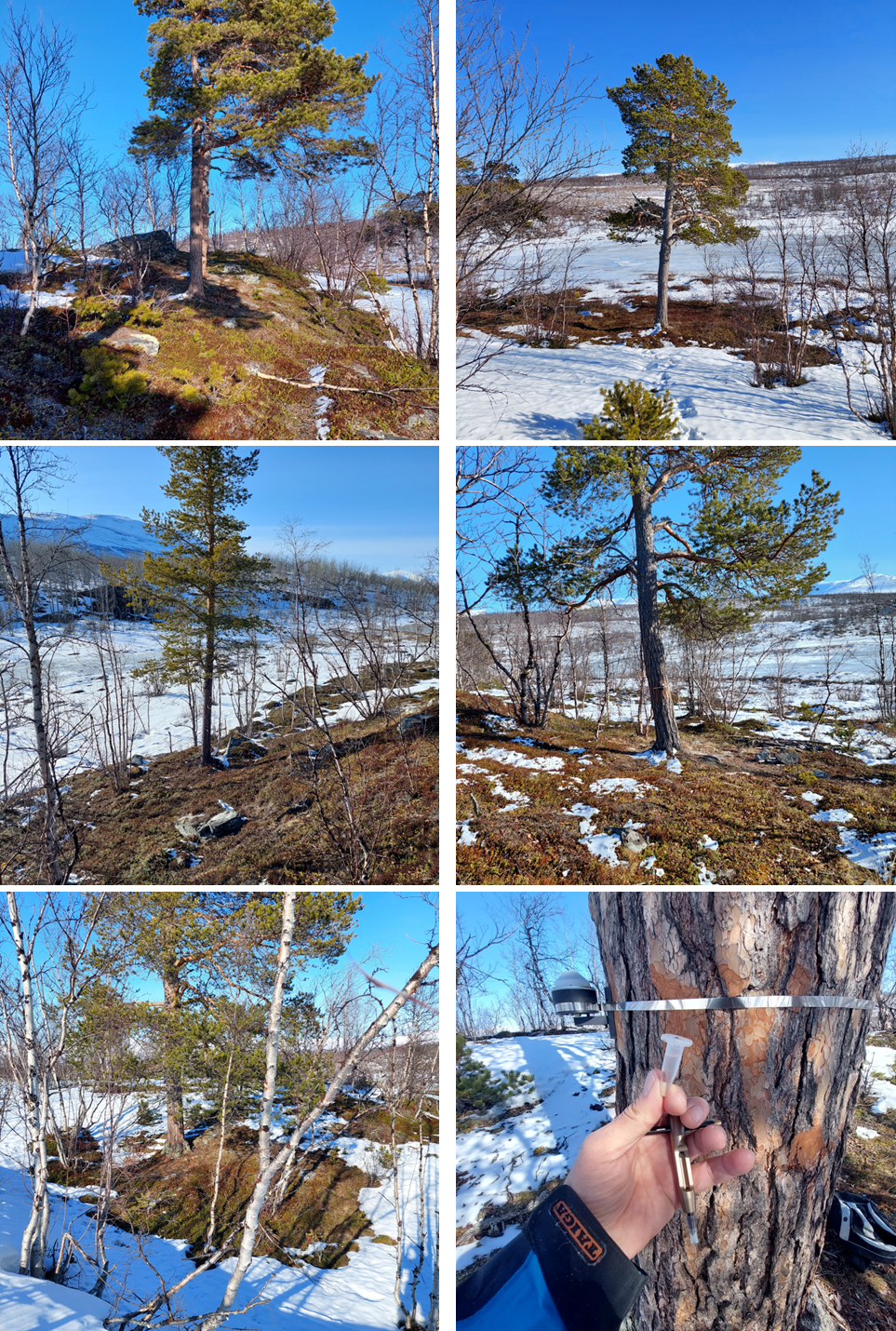
**

**Figure S2**: Overview of the monitored forest stand with dendrometers and the xylogenesis sampling procedure (bottom-right). Pictures were taken by Håkan Grudd on 24^th^ April 2023.


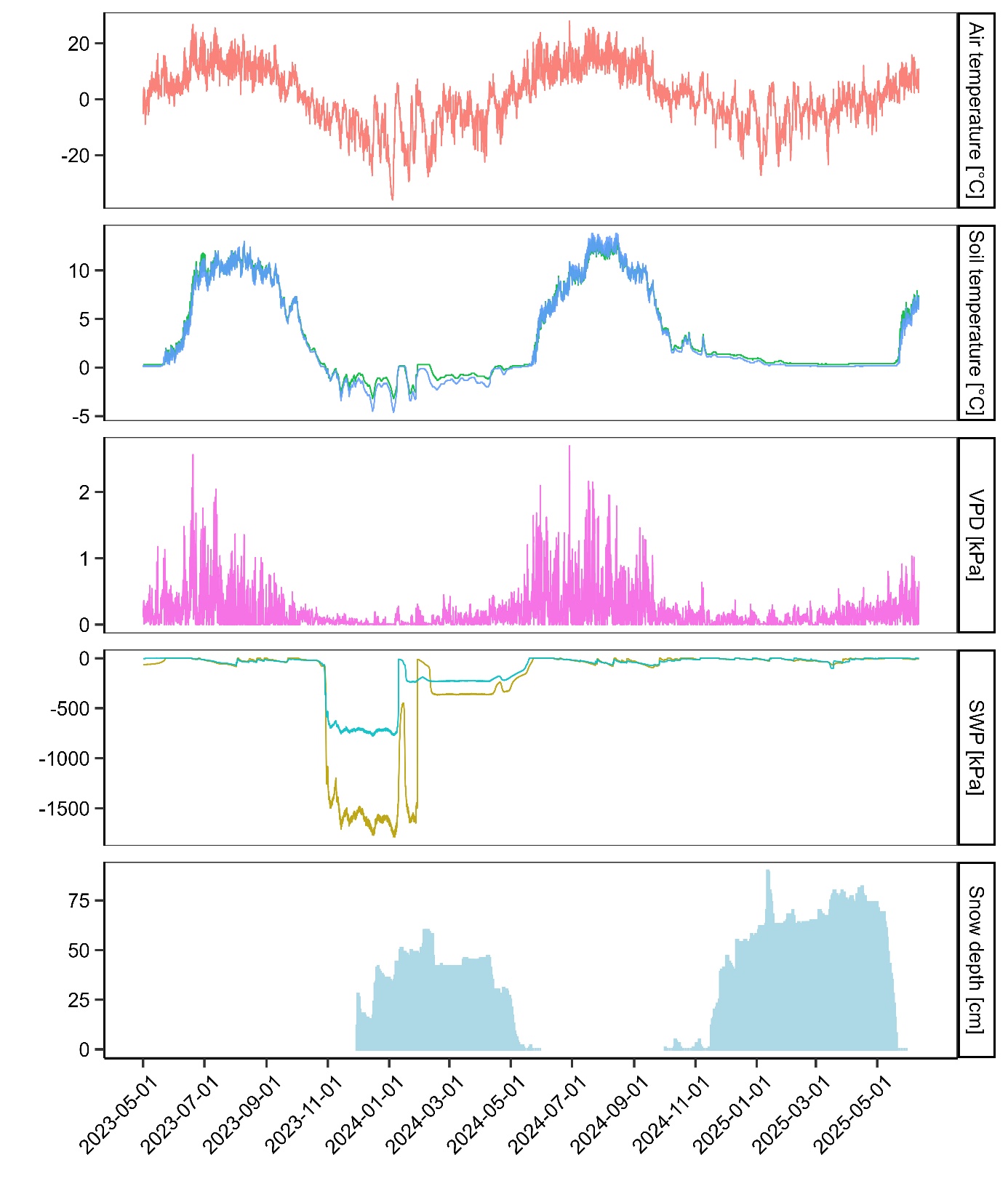


**Figure S3**: Hourly-resolved time series of air and soil meteorological variables measured by automatic sensors at the monitoring site, and daily snow depths measured at the Abisko research station (Source: <https://www.smhi.se/data/hitta-data-for-en-plats/ladda-ner-vaderobservationer/snowDepth/188790>). Soil temperature and soil water potential were recorded by two sensors and averaged before the statistical analysis. Snow data are available only since 30^th^ November 2023. VPD=vapor pressure deficit, SWP=soil water potential.


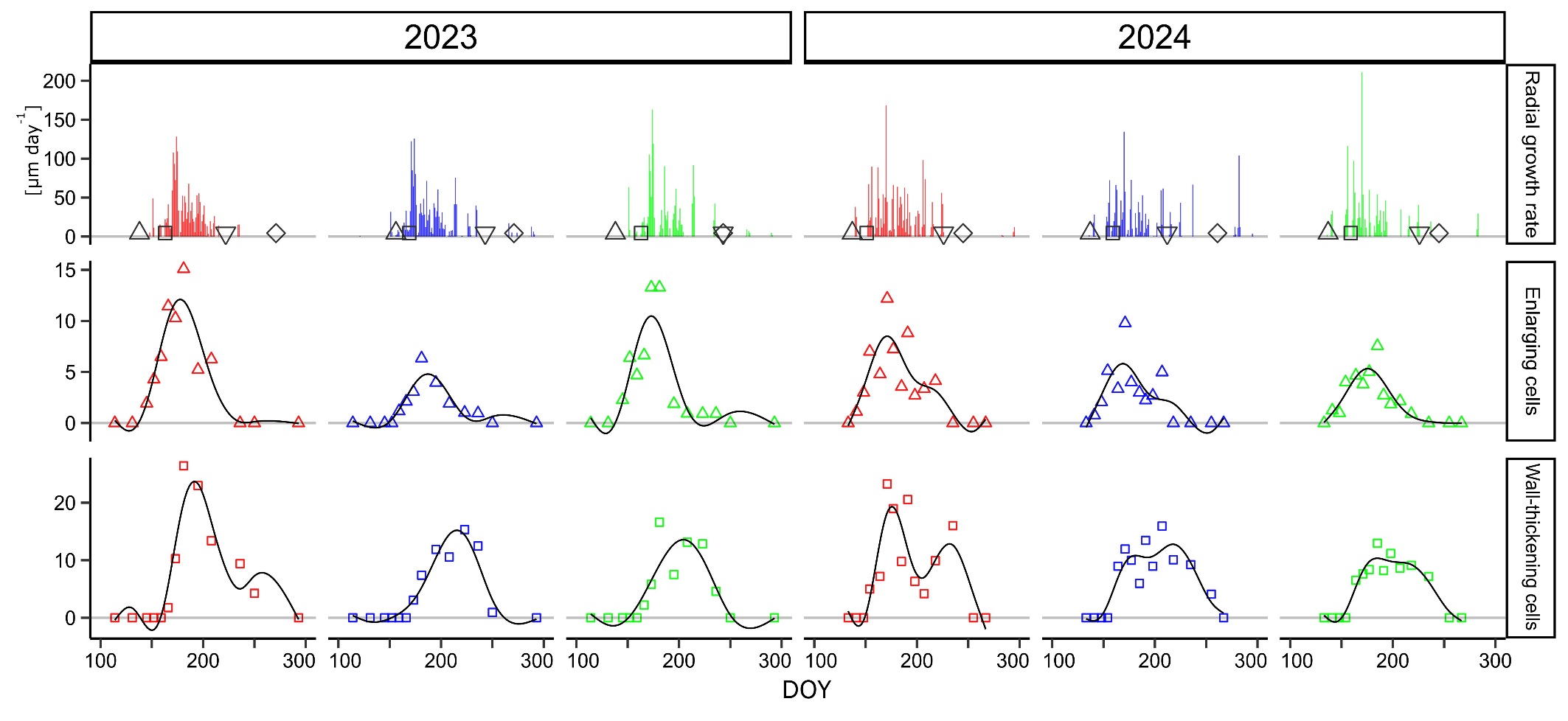


**Figure S4**: Comparison of mean daily-resolved series of radial growth rates recorded by dendrometers (top row) with the number of enlarging (middle row) and wall-thickening (bottom row) cells counted on xylogenesis samples. Colors represent three trees with joint monitoring of intra-annual growth using dendrometers and xylogenesis. Symbols at the bottom of the top charts indicate the mean day for onset and cessation of enlarging (triangles) and wall-thickening (squares) phenological phases derived from xylogenesis records. Points in the xylogenesis series show cell numbers counted on xylogenesis samples on individual sampling dates, and black lines are their smoothed intra-annual trends produced by the generalized additive models. DOY=day of the year.


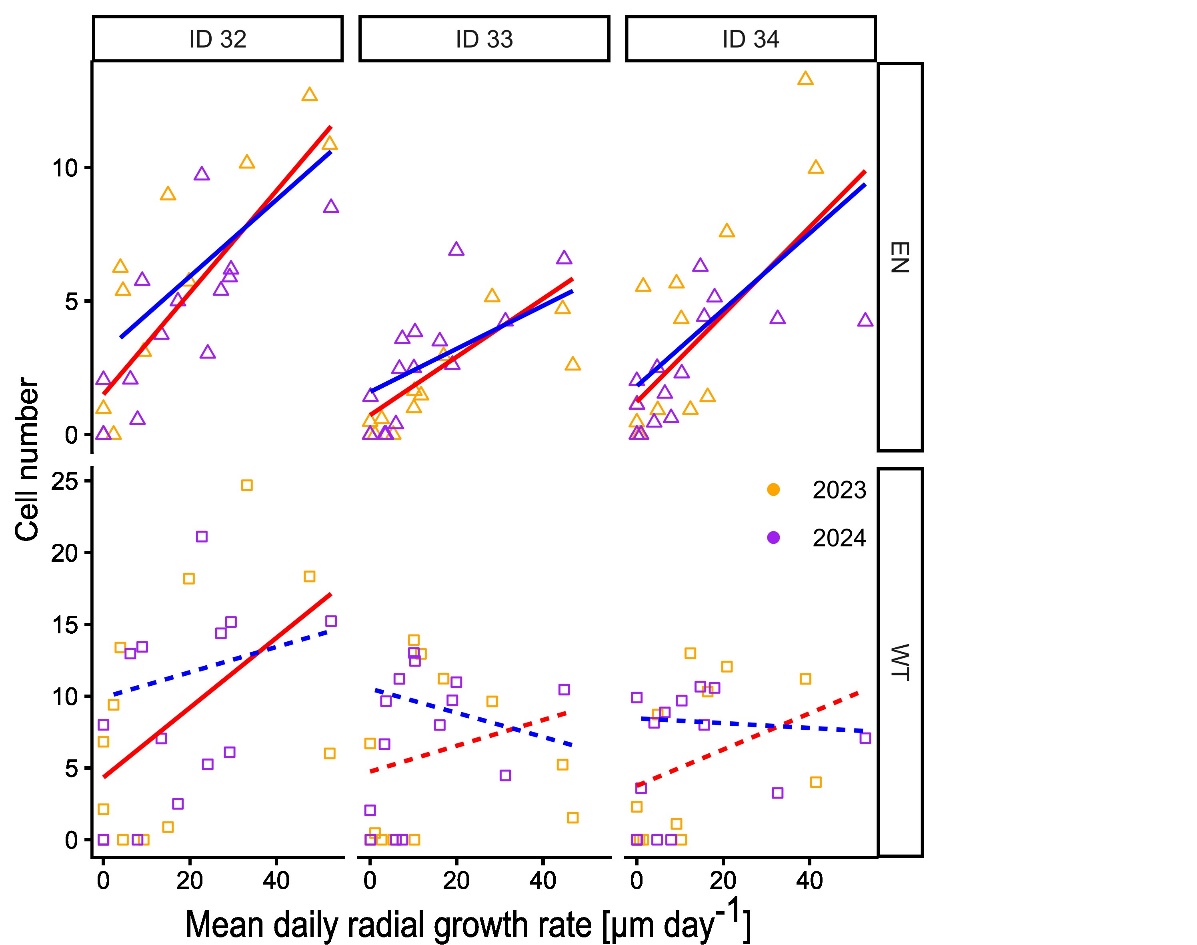


**Figure S5**: Scatterplots between mean daily radial growth rates recorded by dendrometers (x-axes) and mean number of cells (y-axes) in enlargement (EN) and wall-thickening (WT) phases for individual intervals between two consecutive xylogenesis sampling dates. Columns represent three trees with joint monitoring of intra-annual growth by dendrometers and xylogenesis, and the colors of points refer to two years of monitoring. Lines show linear regressions fitted throughout all observations (red) or only observations with non-zero radial growth rate and cell numbers (blue). Solid lines show significant linear regressions (p < 0.05).

**
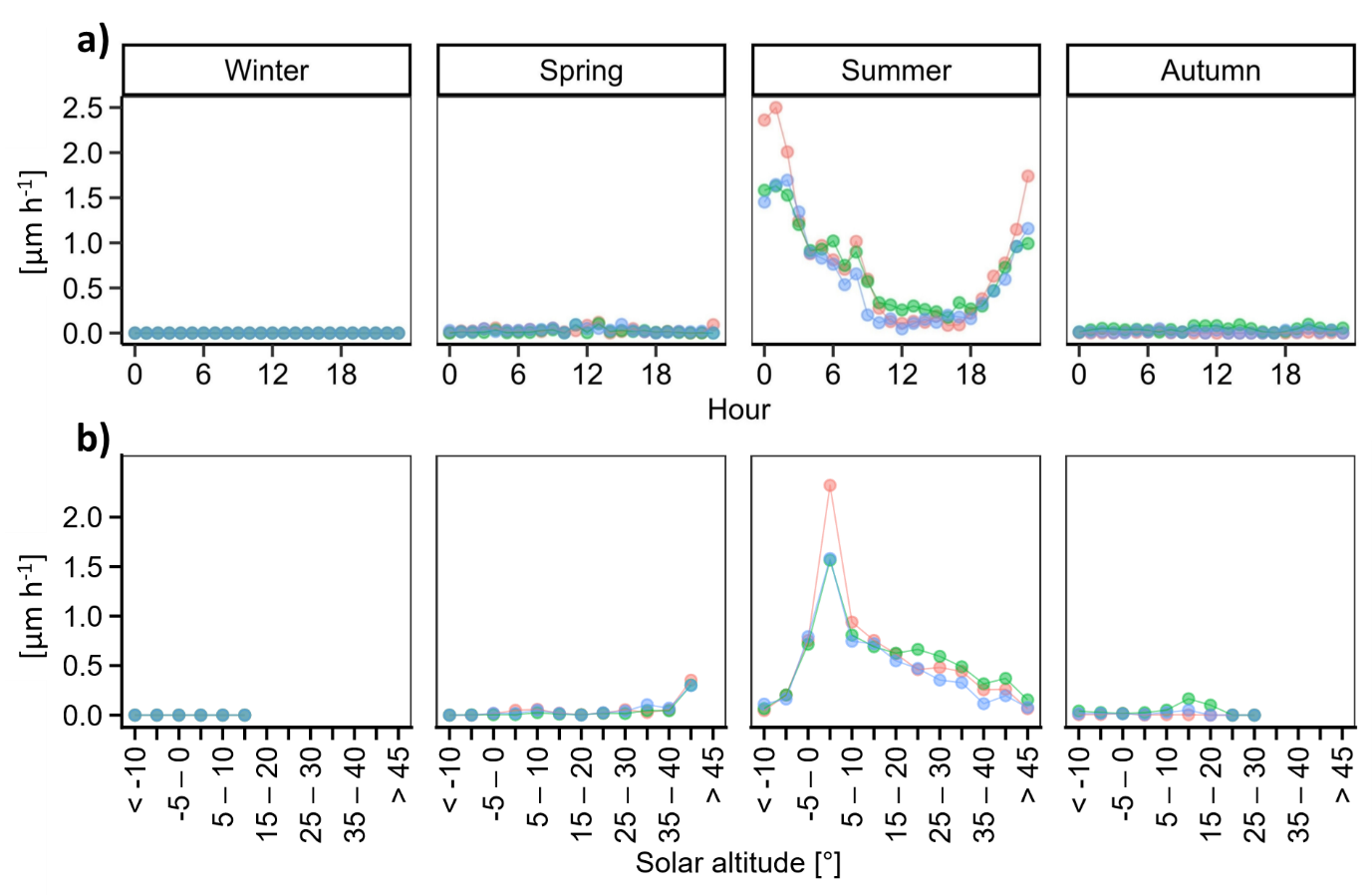
**

**Figure S6**: Mean radial growth rate of three trees (colors) per specific hour of the day and during the four climatological seasons (a). The mean radial growth rate for 5-degree intervals of solar altitude above the horizon during the four climatological seasons (b).


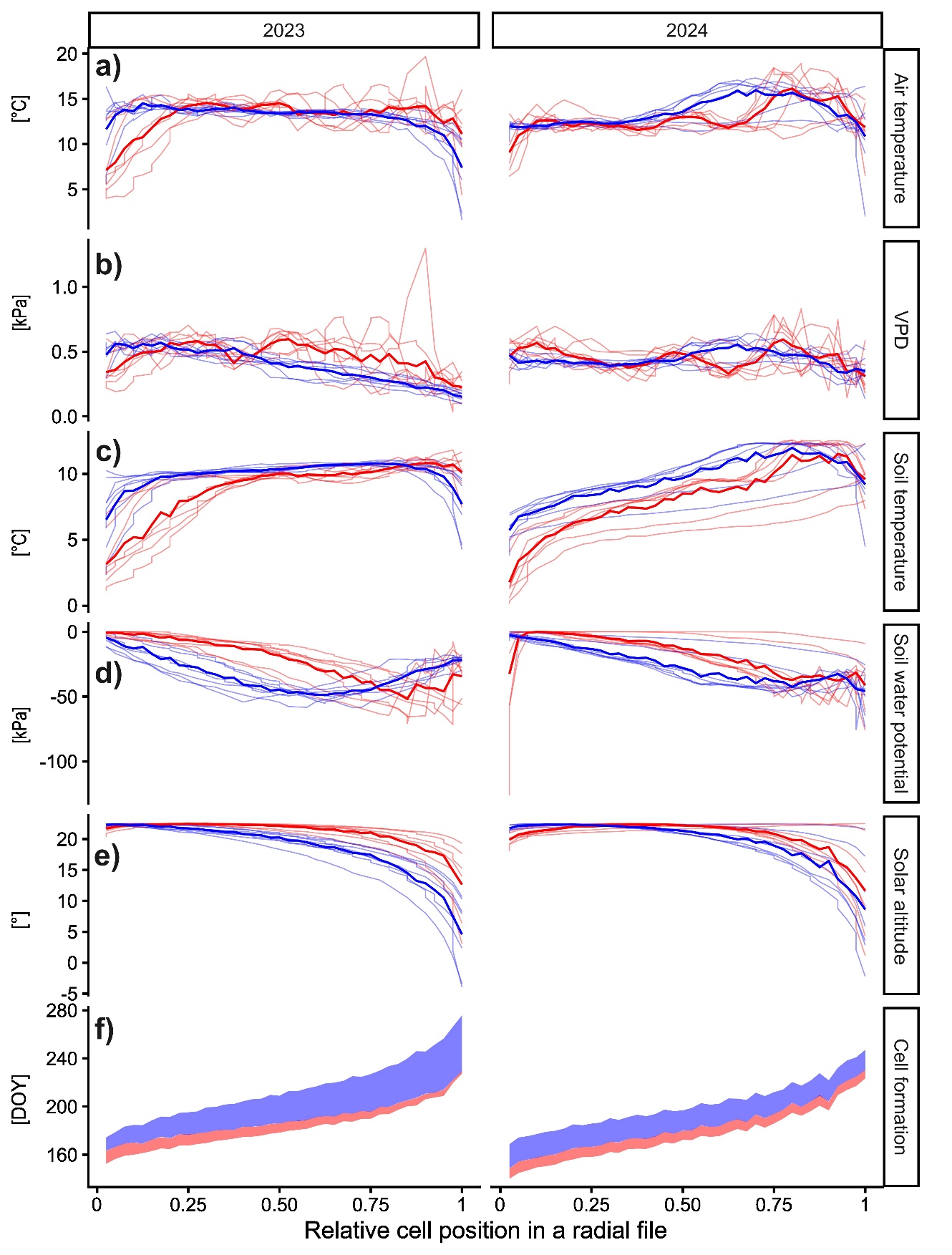


**Figure S7**: Mean meteorological conditions (a-d) and solar altitude (e) experienced by individual cells in a radial file during their lumen enlargement (red) and cell-wall thickening (blue). Timing of lumen enlargement and cell-wall thickening of individual cells during a year (f). Thin lines in (a-e) show data for individual trees; thick lines represent a mean at the site level. DOY = day of the year; VPD = vapor pressure deficit.


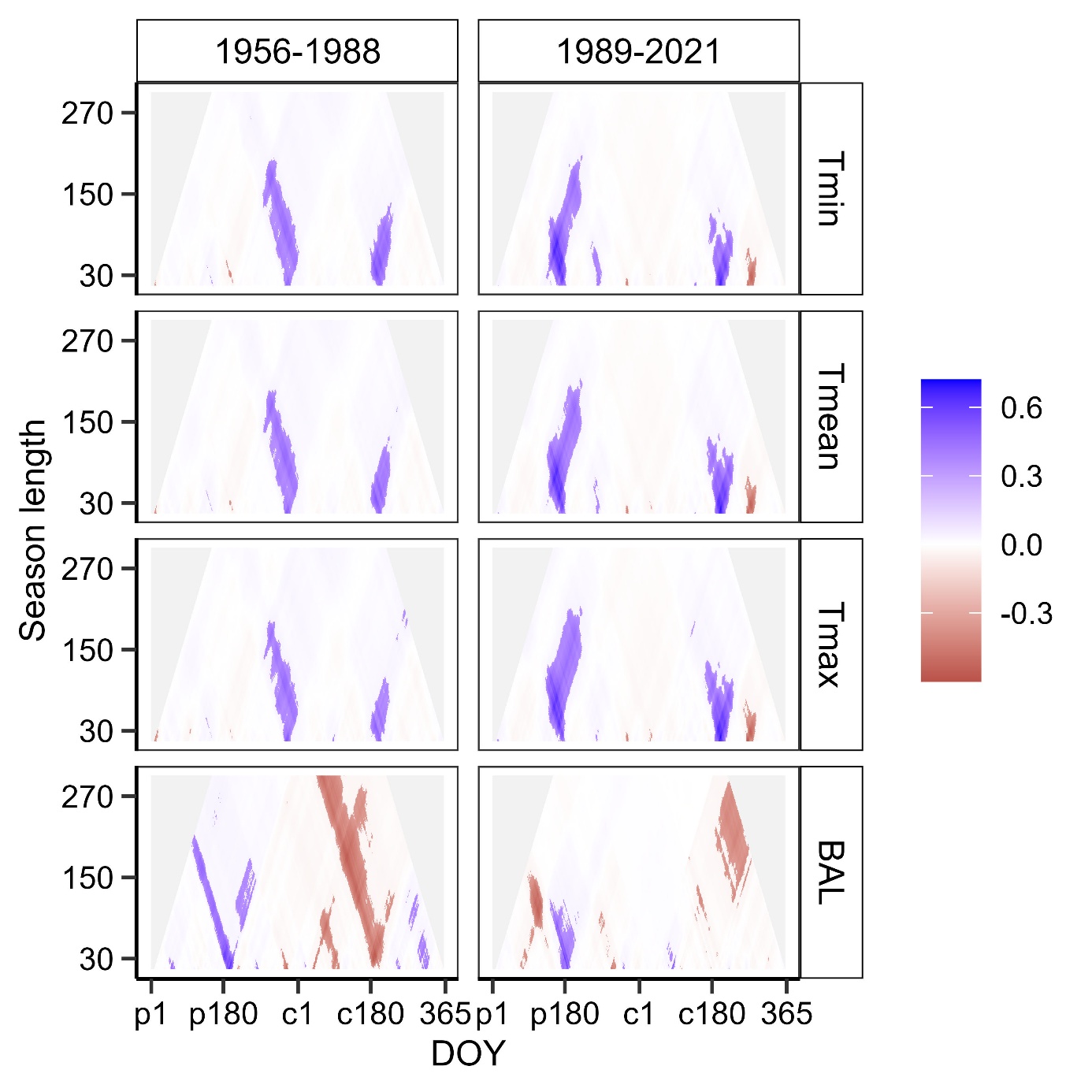


**Figure S8**: Climate-growth correlations between residual tree-ring width chronology from the Torneträsk area located 40 km from our study region and seasonal means of minimum, mean, and maximum temperatures (T) and climatic water balance (BAL) calculated as a difference between daily precipitation and potential evapotranspiration. Climatic series were detrended before calculating correlations. X-axes indicate the center of each season, starting on the first day of the year previous to tree-ring formation (p) until the last day of the year of the tree-ring formation (c). Y-axes indicate the season length from 15 to 300 consecutive days. Colors highlight values of correlation coefficients between chronology and seasonal climatic variables aggregated for the given season, with non-significant values semitransparent (|r|<0.337; p<0.05). Climate-growth correlations were calculated for two independent periods of 33 consecutive years (1956-1988 and 1989-2021) to evaluate shifts in their patterns over time. Although a strong positive effect of current summer temperature on tree-ring width prevailed in both periods, negative impacts of climatic water balance notably weakened over time. Moreover, negative temperature-growth correlations occurred for late-summer seasons in the latter period, while temperature-growth correlations were purely positive in the earlier period. For details about site chronology and its statistical processing, see Tumajer et al. (2025): [10.1016/j.agrformet.2025.110634](https://linkinghub.elsevier.com/retrieve/pii/S0168192325002540) DOY=day of year.

**Table S1**: Outputs of the linear mixed-effects models explaining log-transformed growth rates and binary growth occurrence derived from **dendrometer series** in different temporal resolutions (hour, day, week) using immediate meteorological variables. Predictors represent estimates of slopes of fixed effects and the variance accounted for by random effects. Estimates are shown only for significant predictors (p < 0.05); the others are indicated with n.s. For continuous/binary dependent variables of growth rate/occurrence, the Gaussian/binomial families of models have been used.

| **Temporal resolution** | **Dependent variable** | | **Predictors** | | | | | | **R^2^m** | **R^2^c** |
| --- | --- | --- | --- | --- | --- | --- | --- | --- | --- | --- |
|  |  |  | **Fixed effects - estimates** | | | **Random effects - variance** | | |  |  |
|  |  |  | **VPD** | **SWP** | **Tair:Tsoil** | **(1\|tree)** | **(1\|DOY)** | **(1\|hour)** |  |  |
| Hour | Dendro | Rate | -1.491 | 0.008 | 0.006 | 0.037 | 0.172 | 0.085 | 0.08 | 0.24 |
|  |  | Occurrence | -5.588 | 0.002 | 0.015 | 0.093 | 67.073 | 0.230 | 0.03 | 0.96 |
| Day | Dendro | Rate | -1.926 | 0.018 | 0.010 | 0.037 | 0.751 | NA | 0.12 | 0.47 |
|  |  | Occurrence | -9.942 | 0.007 | 0.44 | 0.108 | 57.765 | NA | 0.13 | 0.95 |
| Week | Dendro | Rate | 3.213 | n.s. | n.s. | 0.011 | NA | NA | 0.30 | 0.30 |
|  |  | Occurrence | 9.186 | 0.017 | 0.008 | 0.058 | NA | NA | 0.94 | 0.94 |

R^2^m=marginal pseudo-R^2^, R^2^c=conditional pseudo-R^2^, VPD=vapor pressure deficit, SWP=soil water potential, Tair=air temperature, Tsoil=soil temperature, DOY=day of year.

**Table S2**: Outputs of the linear mixed-effects models explaining log-transformed cell increments in different temporal resolutions (day, week) simulated from Gompertz functions fitted to **xylogenesis data** using immediate meteorological variables. Predictors represent estimates of slopes of fixed effects and the variance accounted for by random effects. Estimates are shown only for significant predictors (p < 0.05); the others are indicated with n.s.

| **Temporal resolution** | **Dependent variable** | | **Predictors** | | | | | **R^2^m** | **R^2^c** |
| --- | --- | --- | --- | --- | --- | --- | --- | --- | --- |
|  |  |  | **Fixed effects - estimates** | | | **Random effects - variance** | |  |  |
|  |  |  | **VPD** | **SWP** | **Tair:Tsoil** | **(1\|tree)** | **(1\|DOY)** |  |  |
| Day | Xylogenesis | Rate | n.s. | 0.006 | n.s. | 106 | 7830 | 0.01 | 0.79 |
| Week | Xylogenesis | Rate | 72.020 | 0.029 | n.s. | 0 | NA | 0.09 | 0.09 |

R^2^m=marginal pseudo-R^2^, R^2^c=conditional pseudo-R^2^, VPD=vapor pressure deficit, SWP=soil water potential, Tair=air temperature, Tsoil=soil temperature, DOY=day of year.
